# Editorial: Representation in the Brain

**DOI:** 10.3389/fpsyg.2018.01410

**Published:** 2018-08-08

**Authors:** Asim Roy, Leonid Perlovsky, Tarek R. Besold, Juyang Weng, Jonathan C. W. Edwards

**Affiliations:** ^1^Department of Information Systems, Arizona State University, Tempe, AZ, United States; ^2^Department of Psychology, Northeastern University, Boston, MA, United States; ^3^Data Science, City University of London, London, United Kingdom; ^4^Department of Computer Science and Engineering, Michigan State University, East Lansing, MI, United States; ^5^Division of Medicine, University College London, London, United Kingdom

**Keywords:** representation in the brain, localist connectionism, distributed representation, abstract concept encoding, symbolic system

Representation of abstract concepts in the brain at the neural level remains a mystery as we argue over the biological and theoretical feasibility of different forms of representations. We have divided the papers in this special topic on “Representation in the brain” broadly into the following sections:

1. Those arguing, either on a theoretical basis or with neurophysiological evidence, that abstract concepts, simple or complex, exist (have to exist) at the single cell level. Papers by Edwards, Tsotsos, Feldman, and Roy are in this category. However, Feldman and Tsotsos argue that there might be an underlying neural cell assembly (a sub-network) of subconcepts to support a concept at the single cell level. Feldman also stresses action circuits in his paper.
2. There are three papers that argue for sparse distributed representation (population coding) of abstract concepts. Papers by Balkenius and Gärdenfors, Kajic et al., and Lőrincz and Sárkány are in this category.
3. There are two papers discussing neural implementation of symbolic models: one by van der Velde et al. and the other by Wolff.
4. The paper by Frank and Schack, on learning of motor skills from imagery vs. actual execution, is not strictly related to the issue of abstract concept representation, but is about other aspects of learning.

We provide a brief summary of each of the papers next.

## On single cell abstract representation in the brain

Edwards argues that both local and distributed representation is present in the brain and explains which occurs when. He explains that distributed representation occurs on the input side of a neuron, but the neuron itself, being the receiver and interpreter of these signals, is localist. This interpretation of brain architecture essentially resolves the fundamental question of who ultimately establishes meaning and interpretation of a collection of signals. In other words, there has to be a “consumer” (a decoder) of such a collection of signals. Without a “consumer,” the collection of signals is not “received.” In this interpretation, therefore, any signal generated by a neuron has meaning and interpretation. Another neuron, receiving a collection of these signals, then interprets and generates new information. He further argues that this interplay of distributed and localist representation occurs throughout the brain in multiple layers of processing. And he claims that the concept of “representation-as-input” is not in conflict with neuroscience at all.

Tsotsos revisits the issue of complexity analysis, mainly of visual tasks, and claims that complexity analysis, accounting for resource constraints, dictates the type of representation required for visual tasks. He argues that complexity analysis could be used as a test to validate theories of the brain. For example, accounting for the resource constraints, certain computational schemes cannot be feasibly implemented in biological systems. For human vision, such resource constraints include numbers of neurons, synapses, neural transmission times, behavioral response times, and so on. He also examines certain abstract representations in the brain and shows how they reduce problem complexity. For example, certain pyramidal processing structures in the brain (which have origins in the work of Hubel and Wiesel) produce abstract representations and thus reduce the problem size and the search space for algorithms. He quotes Zucker (1981) on the need for explicit abstract representation: “*One of the strongest arguments for having explicit abstract representations is the fact that they provide explanatory terms for otherwise difficult (if not impossible) notions*.” A key conclusion is that knowledge of the intractability of visual processing in the general case tells us that no single solution can be found that is optimal and realizable for all instances. This forces a reframing of the space of all problem instances into sub-spaces where each may be solvable by a different method. This variety of different solution strategies implies that processing resources and algorithms must be dynamically tunable. An executive controller is important to decide among solutions depending on context and to perform this dynamic tuning, and explicit representations must be available to support these functions.

Feldman focuses on brain activity rather than just structure to explain that action and communication are crucial to neural encoding. The paper starts with a brief review of the localist/distributed issue that was active early in the development of connectionist models. He suggests that there is now a consensus—the main mechanism for neural signaling is frequency encoding in functional circuits of low redundancy, often called sparse coding. The main point of the piece is that the term “representation” presupposes a separation of process and data, which is fine for books and computers, but hopeless for the brain. A related point is that brains are not in the storage or truth business, but compute actions and actionability. Actionability is an agent's internal assessment of the expected utility of its possible actions. In addition, the idea of planning, etc. as programs running against data structures should be replaced by mental “simulations.” The final section discusses some mysteries of the mind and suggests that all current theories are incompatible with aspects of our subjective experience. There is evidence for all this, some of which is cited in the short article.

Roy provides extensive evidence for single-cell based simple and complex abstractions from neurophysiological studies of single cells. These single-cell abstractions show up in various forms, but the most significant and complex ones are the category-selective cells, the multisensory neurons and the grandmother-like cells. Category-selective cells encode complex abstract concepts at the highest levels of processing in the brain. There is also extensive evidence for multisensory neurons in the sensory processing areas of the brain. In addition, abstract modality invariant cells (e.g., Jennifer Aniston cells) have been found at higher levels of cortical processing. Overall, according to Roy, these neurophysiology studies reveal the existence of a purely abstract cognitive system in the brain encoded by single cells.

## On sparse distributed representation

Topographic representations are used widely in the brain, such as retinotopy in the visual system, tonotopy in the auditory system and somatotopy in the somatosensory system. These topographic representations are projections from a higher dimensional space (of sensory information) to a lower dimensional one. Such abstract, low-dimensional representations also appear in the entorhinal-hippocampal complex (EHC). Lőrincz and Sárkány introduce the concept of Cartesian Factors (they use it to enable localized discrete representation) and use the concept to model and explain the EHC system. They are Cartesian in the sense that they are like coordinates in an abstract space. And these Cartesian Factors can be used like symbolic variables. They conclude that Cartesian Factors provide a framework for symbol formation, symbol manipulation, and symbol grounding processes at the cognitive level.

In Remote Associates Test (RAT), subjects are presented with three cue words (e.g., *fish, mine*, and *rush*) and have to find a solution word (e.g., *gold*) related to all cues within a time limit. RAT is commonly used to find an individual's ability to think creatively and finding a novel solution word is usually associated with creativity. Kajic et al. present a spiking neuron model for RAT. Their model shows significant correlation with human performance on such a task. They use distributed representation in their model, but each neuron in such a representation has a preferred stimuli similar to what is found in the visual system and place cells. They used leaky integrate-and-fire spiking neurons in the model. Their RAT model is the first one to link such a cognitive process with neural implementation. However, their current model does not explain how humans learn such word associations. All connection weights and other parameters were determined in an offline mode.

Humans and animals use abstractions (information compression) at different levels of processing in the brain. For example, cones and rods in the retina code for 3-dimensional color perception in humans. Such abstractions to lower dimensional spaces occur explicitly throughout sensory systems. Balkenius and Gärdenfors a, in their paper explain how the brain can abstract from neurocognitive representations to psychological spaces and show how population coding at the neural level can generate these abstractions. They show that radial basis function networks are ideal structures for mapping population codes to such lower dimensional spaces. In their theory, the coding of the low-dimensional spaces need not be explicitly expressed in individual neurons but the spatial structures are emergent properties. They also argue that the mediation between perception and action occurs through such spatial representations and that this form of mediation results in more efficient learning.

## Neural implementations of symbolic models

van der Velde et al. explore the characteristics of two architectures for representing and processing complex conceptual (sentence-like) structures: (1) the Neural Blackboard Architecture (NBA), which is at the neural level, and (2) the Information Dynamics of Thinking (IDyOT) architecture, which is at the symbolic level. They then explore the combination of these two architectures for the purpose of creating both an artificial cognitive system and to explain representation and processing of such structures in the brain. With IDyOT, one can learn the structural elements from real corpora. NBA provides a way to neurally implement IDyOT, whereas IDyOT itself provides a higher-level formal account and learning abilities. Overall, the combined architecture provides a connection between neural and symbolic levels.

Wolff outlines how his “SP Theory of Intelligence” (where “SP” stands for *Simplicity* and *Power*), can be implemented using connected neurons and signal transmission between them. He calls this neural extension “SP-neural”. In the SP theory different kinds of knowledge are represented with *patterns*, where a pattern is an array of atomic symbols in one or two dimensions. In SP-neural, these patterns are realized using an array of neurons, a concept similar to Hebb's cell assembly, but with important differences. The central concept in the SP theory is information compression via “SP-multiple-alignment.” A favorable combination of Simplicity and Power is aimed for by trying to maximize compression. In the SP theory, unsupervised learning is the basis for other kinds of learning—supervised, reinforcement, imitation and so on.

## Learning from imagery vs. execution

Frank and Schack provide an overview of the literature on learning of motor skills by imagery and execution from three different perspectives—performance (actual changes in motor behavior), the brain (changes in the neurophysiological representation of motor action) and the mind (changes in the perceptual-cognitive representation of motor action). Both simulation and execution of motor action leads to functional changes in the motor action system through learning, although perhaps to a different extent. They observe, however, that very little is known about how actual learning takes place under these different forms of motor skill practice, especially in terms of action representation.

## Author contributions

AR summarized the topic articles with contributions from LP, TB, JW, and JE.

### Conflict of interest statement

The authors declare that the research was conducted in the absence of any commercial or financial relationships that could be construed as a potential conflict of interest.

## References

[B1] Zucker S. W. (1981). Computer vision and human perception: an essay on the discovery of constraints, in Proceedings 7th International Conference on Artificial Intelligence, eds P. Hayes and R. Schank (Vancouver, BC), 1102–1116.

